# A randomised phase 2a study to investigate the effects of blocking interleukin-33 with tozorakimab in patients hospitalised with COVID-19: ACCORD-2

**DOI:** 10.1183/23120541.00249-2023

**Published:** 2023-10-02

**Authors:** Tom Wilkinson, Anthony De Soyza, Miles Carroll, James D. Chalmers, Michael G. Crooks, Gareth Griffiths, Manu Shankar-Hari, Ling-Pei Ho, Alex Horsley, Chris Kell, Beatriz Lara, Biswa Mishra, Rachel Moate, Clive Page, Hitesh Pandya, Jason Raw, Fred Reid, Dinesh Saralaya, Ian C. Scott, Salman Siddiqui, Andy Ustianowski, Natalie van Zuydam, Ashley Woodcock, Dave Singh

**Affiliations:** 1 NIHR Southampton Biomedical Research Centre and University of Southampton, Southampton, UK; 2 Population Health Sciences Institute, Faculty of Medical Sciences, Newcastle University, Newcastle upon Tyne, UK; 3 Pandemic Sciences Institute, Nuffield Department of Medicine, University of Oxford, Oxford, UK; 4 Division of Molecular and Clinical Medicine, University of Dundee, Ninewells Hospital and Medical School, Dundee, UK; 5 Hull York Medical School, University of Hull, Hull, UK; 6 Southampton Clinical Trials Unit, University of Southampton, Southampton, UK; 7 University Hospital Southampton NHS Foundation Trust, Southampton, UK; 8 Centre for Inflammation Research, University of Edinburgh, Edinburgh, UK; 9 Medical Research Council Human Immunology Unit, University of Oxford, Oxford, UK; 10 Division of Immunology, Immunity to Infection and Respiratory Medicine, School of Biological Sciences, University of Manchester, Manchester, UK; 11 Research and Early Development, Respiratory and Immunology, BioPharmaceuticals R&D, AstraZeneca, Cambridge, UK; 12 University Hospitals Coventry and Warwickshire NHS Trust, Coventry, UK; 13 Royal Oldham Hospital, Oldham, UK; 14 Early Biometrics, AstraZeneca, Cambridge, UK; 15 Sackler Institute of Pulmonary Pharmacology, King's College London, London, UK; 16 Clinical Development, Research and Early Development, Respiratory and Immunology, BioPharmaceuticals R&D, AstraZeneca, Cambridge, UK; 17 Fairfield Hospital, Bury, UK; 18 Bradford Teaching Hospitals NHS Foundation Trust, Bradford, UK; 19 Translational Science and Experimental Medicine, Research and Early Development, Respiratory and Immunology, BioPharmaceuticals R&D, AstraZeneca, Cambridge, UK; 20 Faculty of Medicine, National Heart and Lung Institute, Imperial College London, London, UK; 21 Regional Infection Unit, North Manchester General Hospital, Manchester, UK; 22 Discovery Sciences, AstraZeneca, Gothenburg, Sweden; 23 Medicines Evaluation Unit, Manchester University NHS Foundation Trust, Manchester, UK

## Abstract

**Background:**

Increased serum interleukin (IL)-33 predicts poor outcomes in patients hospitalised with coronavirus disease 2019 (COVID-19). We examined the efficacy and safety of tozorakimab, a monoclonal antibody that neutralises IL-33, in improving outcomes in ACCORD-2 (EudraCT: 2020-001736-95).

**Methods:**

ACCORD-2 was an open-label, phase 2a study in adults hospitalised with COVID-19. Patients were randomised 1:1 to tozorakimab 300 mg plus standard of care (SoC) or SoC alone. The primary end-point was time to clinical response (sustained clinical improvement of ≥2 points on the World Health Organization ordinal scale, discharge from hospital or fit for discharge) by day 29. Other end-points included death or respiratory failure, mortality and intensive care unit admission by day 29, and safety. Serum IL-33/soluble stimulated-2 (sST2) complex levels were measured by high-sensitivity immunoassay.

**Results:**

Efficacy analyses included 97 patients (tozorakimab+SoC, n=53; SoC, n=44). Median time to clinical response did not differ between the tozorakimab and SoC arms (8.0 and 9.5 days, respectively; HR 0.96, 80% CI 0.70–1.31; one-sided p=0.33). Tozorakimab was well tolerated and the OR for risk of death or respiratory failure with treatment *versus* SoC was 0.55 (80% CI 0.27–1.12; p=0.26), while the OR was 0.31 (80% CI 0.09–1.06) in patents with high baseline serum IL-33/sST2 complex levels.

**Conclusions:**

Overall, ACCORD-2 results suggest that tozorakimab could be a novel therapy for patients hospitalised with COVID-19, warranting further investigation in confirmatory phase 3 studies.

## Tweetable abstract

**Tozorakimab was well tolerated, and *post hoc* analyses suggest that treatment effects may be enhanced in patients with severe COVID-19 and those with high baseline levels of serum IL-33/sST2. These effects will be investigated in a global phase 3 study.** https://bit.ly/3YtME1M

## Introduction

Coronavirus disease 2019 (COVID-19) has rapidly developed into a global health threat [1]. The pathogenesis of severe COVID-19 is driven by complex immuno-inflammatory dysregulation [2, 3]. This dysregulation may lead to acute respiratory distress and multiorgan failure [3–6] ∼7 days after the first symptoms [2].

Few approved therapeutic agents are currently available to treat severe COVID-19, and despite the impact of vaccination on reducing severe disease and mortality, there remains an urgent need for the rapid development of efficacious interventions. Immunocompromised or unvaccinated individuals remain at risk of severe disease [7–11], and vaccinated individuals carry a residual risk because vaccines are <100% effective and reduce in effectiveness over time owing to waning immunity and the emergence of new variants [7–9]. Knowledge of prognostic biomarkers to identify patients at risk of poor outcomes and predictive biomarkers to identify responders to therapeutic agents would aid the development of novel drugs [12].

Interleukin (IL)-33 is a broad-acting epithelial “alarmin” cytokine constitutively expressed and stored in epithelial and endothelial cells [13], where it is rapidly released in response to cellular stress, tissue injury or infection [14]. Reduced IL-33 signals *via* serum stimulated-2 (ST2), whereas oxidised IL-33 signals *via* a complex of receptors for advanced glycation end-products and epidermal growth factor [15, 16]. ST2 is expressed in two isoforms, cell surface receptor ST2L and soluble ST2 (sST2), which is an endogenous antagonist of IL-33 activities [17, 18]. Excess release of IL-33 may drive dysregulated hyper-inflammation in severe acute respiratory syndrome coronavirus 2 infection [19]; IL-33 levels are increased in patients with COVID-19 and are associated with disease severity [20]. Tozorakimab is a high-affinity human IgG1 monoclonal antibody that neutralises IL-33 [15] and has therapeutic potential to improve clinical outcomes in patients hospitalised with COVID-19.

ACCORD-2 is a randomised, adaptive-platform, phase 2a study designed for the rapid assessment of multiple treatments added to standard of care (SoC) for patients hospitalised with COVID-19. The protocol of this study has been published previously [21]. The study was designed as a master protocol with candidate drugs evaluated using subprotocols [21]. The aim of the subprotocol presently discussed was to evaluate the efficacy and safety of tozorakimab in improving clinical outcomes in patients hospitalised with COVID-19.

## Methods

### Study participants

The study included patients aged ≥18 years, who were hospitalised with COVID-19 and met the clinical status of grade 3 (hospitalised; mild disease, no oxygen therapy), 4 (hospitalised; oxygen by mask or nasal prongs) or 5 (hospitalised; non-invasive ventilation or high-flow oxygen) of the World Health Organization Working Group on the Clinical Characteristics of COVID-19 9-point ordinal scale (OS) 2020, as per the study protocol [21]. All patients provided written informed consent. Key exclusion criteria included a previous score of grade 6 or 7 on the OS, myocardial infarction in the 3 months before the first dose of study treatment, unstable angina, a history of clinically significant arrhythmia, stage 4 chronic kidney disease or requiring dialysis. Exclusion criteria specific to the tozorakimab subprotocol were patients with active tuberculosis and those with a known family history of heart failure. Full inclusion and exclusion criteria are listed in the supplementary material.

### Study design

The randomised, open-label, seamless, adaptive, controlled, phase 2 study was conducted in over 15 centres in the UK. The study was initially planned as a two-part study: stage 1 being the pilot stage and stage 2 being the confirmatory stage. Stage 1 was planned to assess the following: preliminary safety and efficacy, optimal study end-points, and the number of patients to enrol in stage 2 of the study. Following changes in COVID-19 presentation, only the first part was conducted, the results of which are reported in this publication. The study was associated with the UK COVID-19 Antivirals and Therapeutics Taskforce, designed to evaluate potential treatments for COVID-19. Patients were recruited during two periods: 20 May 2020 to 24 July 2020 (period 1) and 8 December 2020 to 2 March 2021 (period 2). These periods coincided with COVID-19 waves that occurred in the UK. Period 1 occurred when no vaccinations were available. The B.1.1.7 virus, which was more virulent than previous COVID-19 variants [22], emerged during period 2. The study assessed the efficacy and safety of agents added to SoC *versus* SoC alone.

The study protocol was reviewed and approved by the UK Medicines and Healthcare Products Regulatory Agency (EudraCT: 2020-001736-95; registered 28 April 2020). Ethical approval was received from the relevant Health Research Authority and Research Ethics Committee. An independent data monitoring committee assessed safety throughout the study. The study was conducted in accordance with the ethical principles of the Declaration of Helsinki, Council for International Organizations of Medical Sciences International Ethical Guidelines, International Council for Harmonisation (ICH) Good Clinical Practice Guidelines, and applicable local laws and regulations. The ACCORD-2 study was sponsored by University Hospital Southampton NHS Foundation Trust, and was funded by UK Research and Innovation.

### Drug administration

Patients were randomised to receive either a single dose of tozorakimab 300 mg intravenously in addition to SoC (tozorakimab arm) or SoC alone (SoC arm). Tozorakimab was provided by AstraZeneca. Patients started treatment upon randomisation (day 1), within 24 h of enrolment and screening (see supplementary material for details). A second dose of tozorakimab 300 mg was administered on day 15 if the patient was receiving invasive mechanical ventilation. The tozorakimab dose rationale for patients with COVID-19 was based on results of a tozorakimab phase 1 study in healthy volunteers and patients with COPD [23, 24]. The SoC was based on appropriate guidelines in place at the time of each patient's participation and it consequently evolved over time. Agents administered as SoC are listed in the supplementary material.

### Outcomes

The primary end-point was time from randomisation to clinical response, defined as sustained clinical improvement of ≥2 points on the OS, discharge from hospital or considered fit for discharge (0, 1 or 2 on the OS), whichever came first, by day 29. Sustained clinical improvement was defined as improvement without subsequent worsening before day 29. Patients who did not meet the aforementioned conditions by day 29 were censored at the day of their last OS assessment (*i.e.* either on day 29 or earlier), except for patients who died before day 29, who were censored at day 29.

Secondary end-points assessed were death or respiratory failure (according to the OS) at day 29; survival (mortality at days 15, 29 and 60); proportion of patients not deteriorating according to the OS by 1, 2 or 3 points on days 15 and 29; duration of new invasive ventilation and duration of ventilation-free days; and duration of intensive care unit (ICU) stay and hospitalisation. Safety was also assessed as a secondary end-point (see supplementary material for further details of safety assessments).

### Exploratory biomarker analysis

The secondary outcome of risk of death or respiratory failure at day 29 was assessed by baseline levels of the serum biomarker IL-33/sST2 complex and by median baseline serum sST2 levels. It was hypothesised that a high level of baseline IL-33/sST2 complex may be a potential predictive biomarker to identify tozorakimab responders. Increased serum sST2 levels have been shown to be a prognostic marker of poor outcomes in patients with COVID-19 [25]. The level of serum IL-33/sST2 complex was measured by high-sensitivity immunoassay (S-PLEX; Meso Scale Diagnostics, Rockville, MD, USA) [26]. Serum sST2 was measured using the Presage sST2 immunoassay (Critical Diagnostics, San Diego, CA, USA).

### Statistical analyses

The prespecified significance threshold for assessing efficacy in this initial pilot stage of the study was a 10% one-sided level. This was considered appropriate for identifying potential signals of efficacy for further evaluation in the confirmatory stage. It was expected that 54 patients per arm would provide 80% power to detect a HR of 1.6 for the primary end-point when comparing each candidate agent with SoC, assuming 70% of patients in the SoC arm would improve, be discharged from hospital or be considered fit for discharge at day 29.

The safety analysis set included all patients who received at least one dose of study medication, and was used for presentation of baseline and safety data. The full analysis set, used for presentation of efficacy data, included all patients who received at least one dose of study medication and for whom at least one post-baseline OS score was available.

Primary end-point data were analysed using a log-rank test. The hazard ratio and associated two-sided 80% confidence interval representing the overall treatment effect were estimated using the stratified Cox proportional hazards regression model, containing treatment, age and baseline severity grade as covariates. The median, quartiles and two-sided 80% confidence intervals were estimated using the Kaplan–Meier method. For secondary end-points, proportions were analysed using the Cochran–Mantel–Haenszel test (stratified by baseline severity grade), with odds ratio and 80% Wald confidence intervals calculated using logistic regression, adjusting for age and baseline severity grade.

Outcomes were presented for the study overall; however, owing to differences in COVID-19 variants and changes in SoC during the study, additional *post hoc* exploratory analyses of key end-points were reported for patients recruited during period 2 only. The exploratory subgroup analyses were conducted for the following biomarkers with cut-offs determined by the overall median value at baseline: IL-33/sST2 complex (<30.15 or ≥30.15 U·mL^−1^) and sST2 (<114.6 or ≥114.6 ng·mL^−1^).

All analyses were reported according to the ICH E9 guidelines on statistical principles in clinical trials. Further methodology details are available in the supplementary material.

## Results

### Patient characteristics

**FIGURE 1:**
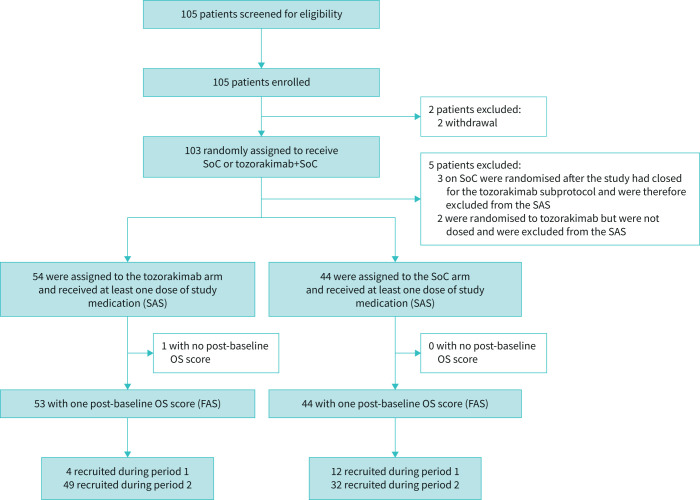
Trial profile. SoC: standard of care; SAS: safety analysis set; OS: ordinal scale; FAS: full analysis set.

Overall, 105 patients were enrolled in the tozorakimab subprotocol and 103 were randomised. Five patients were excluded from the safety analysis set (n=98: tozorakimab+SoC, n=54; SoC alone, n=44) (figure 1). Most patients were recruited during period 2 (tozorakimab+SoC, n=49; SoC alone, n=32). One patient in the tozorakimab arm did not have a post-dose OS assessment and was excluded from the full analysis set (n=97) but was included in the safety analysis set (n=98).

Baseline demographics and patient characteristics of the overall cohort and of those recruited during period 2 are reported in table 1 and supplementary table S1, respectively. In the overall cohort, the mean±sd age was 55.4±12.5 and 58.0±13.9 years in the tozorakimab and SoC arms, respectively (table 1). Patients in the tozorakimab arm had a higher mean±sd baseline National Early Warning Score 2 (NEWS2) score than those in the SoC arm (4.8±2.2 *versus* 4.0±2.1, respectively) (table 1). More patients in the tozorakimab arm than in the SoC arm had at least two comorbidities (38.9% *versus* 29.5%, respectively) (table 1). Most patients had a grade 4 clinical status (77.8% and 75.0% in the tozorakimab and SoC arms, respectively). One patient in the SoC arm (2.3%) was vaccinated at baseline; additional patients were vaccinated during the study (n=13 (24.1%) and n=4 (9.1%) in the tozorakimab and SoC arms, respectively).

**TABLE 1:** Baseline demographics and patient characteristics

	**Tozorakimab+SoC** **(n=54)**	**SoC** **(n=44)**
**Age, years**	55.4±12.5	58.0±13.9
≥70 years	8 (14.8)	11 (25.0)
**Male**	37 (68.5)	29 (65.9)
**BMI, kg·m^−2^**	32.6±8.1	33.3±8.5
**Smoking status**		
Ex-smoker	26 (48.1)	16 (37.2)
Current smoker	2 (3.7)	0 (0.0)
**Time since onset of symptoms**		
<12 days	38 (70.4)	37 (84.1)
≥12 days	16 (29.6)	7 (15.9)
**Derived baseline WHO OS score**		
Grade 3	1 (1.9)	3 (6.8)
Grade 4	42 (77.8)	33 (75.0)
Grade 5	11 (20.4)	8 (18.2)
**NEWS2 score**	4.8±2.2	4.0±2.1
**Clinical frailty score at baseline**		
Very fit	8 (14.8)	4 (9.1)
Well	22 (40.7)	16 (36.4)
Managing well	8 (14.8)	6 (13.6)
Vulnerable	6 (11.1)	5 (11.4)
Mildly frail	3 (5.6)	4 (9.1)
Moderately frail	7 (13.0)	9 (20.5)
**Comorbidities at baseline, n** **^#^**		
≥1	34 (63.0)	25 (56.8)
≥2	21 (38.9)	13 (29.5)
**Comorbidity categories** **^#^**		
Heart disease	5 (9.3)	6 (13.6)
Diabetes	22 (40.7)	13 (29.5)
Chronic lung disease	7 (13.0)	4 (9.1)
Chronic liver disease	1 (1.9)	0 (0.0)
Asthma	8 (14.8)	7 (15.9)
HIV	0 (0.0)	0 (0.0)
Tuberculosis	0 (0.0)	1 (2.3)
Cancer^¶^	5 (9.6)	1 (2.4)
Hypertension^¶^	15 (28.8)	13 (31.7)
**Remdesivir at baseline** **^¶^**	34 (65.4)	25 (61.0)
**Dexamethasone at baseline** **^¶^**	52 (100.0)	37 (90.2)
**Supplemental oxygen at baseline**	53 (98.1)	41 (93.2)
**Received COVID-19 vaccine during the study**	13 (24.1)	4 (9.1)

Data are presented as mean±sd or n (%). SoC: standard of care; BMI: body mass index; WHO: World Health Organization; OS: ordinal scale; NEWS2: National Early Warning Score 2; COVID-19: coronavirus disease 2019. ^#^: percentage values are based on the safety analysis set; ^¶^: percentage values are based on 52 patients receiving tozorakimab+SoC and 41 patients receiving SoC.

### Primary outcome

**FIGURE 2:**
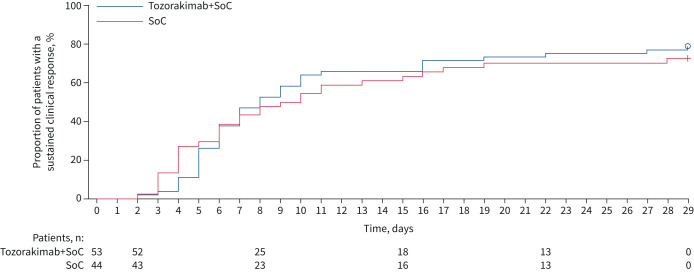
Kaplan–Meier analysis of time to sustained clinical response by day 29. Kaplan–Meier curves were compared using a stratified log-rank test. SoC: standard of care.

The median (interquartile range) time to clinical response by day 29 did not differ between the two arms (tozorakimab arm 8.0 (5.0–22.0) days; SoC arm 9.5 (4.0–not estimable) days; HR 0.96, 80% CI 0.70–1.31; one-sided p=0.33) (figure 2 and table 2).

**TABLE 2:** Primary end-point: time to sustained clinical response

	**Overall**		**Period 2 only^#^**	
	**Tozorakimab+SoC** **(n=53)**	**SoC** **(n=44)**	**Tozorakimab+SoC** **(n=49)**	**SoC** **(n=32)**
**Patients with a sustained clinical response**	42 (79.2)	32 (72.7)	39 (79.6)	22 (68.8)
**Censored**	11 (20.8)	12 (27.3)	10 (20.4)	10 (31.3)
**Time to response, days**	8.0 (5.0–22.0)	9.5 (4.0–NE)	8.0 (5.0–22.0)	8.5 (54.5–NE)
**HR (80% CI); p-value**	0.96 (0.70–1.31); 0.33		1.09 (0.77–1.54)	

Data are presented as n (%) or median (interquartile range), unless otherwise stated. SoC: standard of care; NE: not estimable. ^#^: data for period 1 were not analysed separately owing to the very small proportion of patients recruited in period 1.

### Secondary outcomes

**FIGURE 3:**
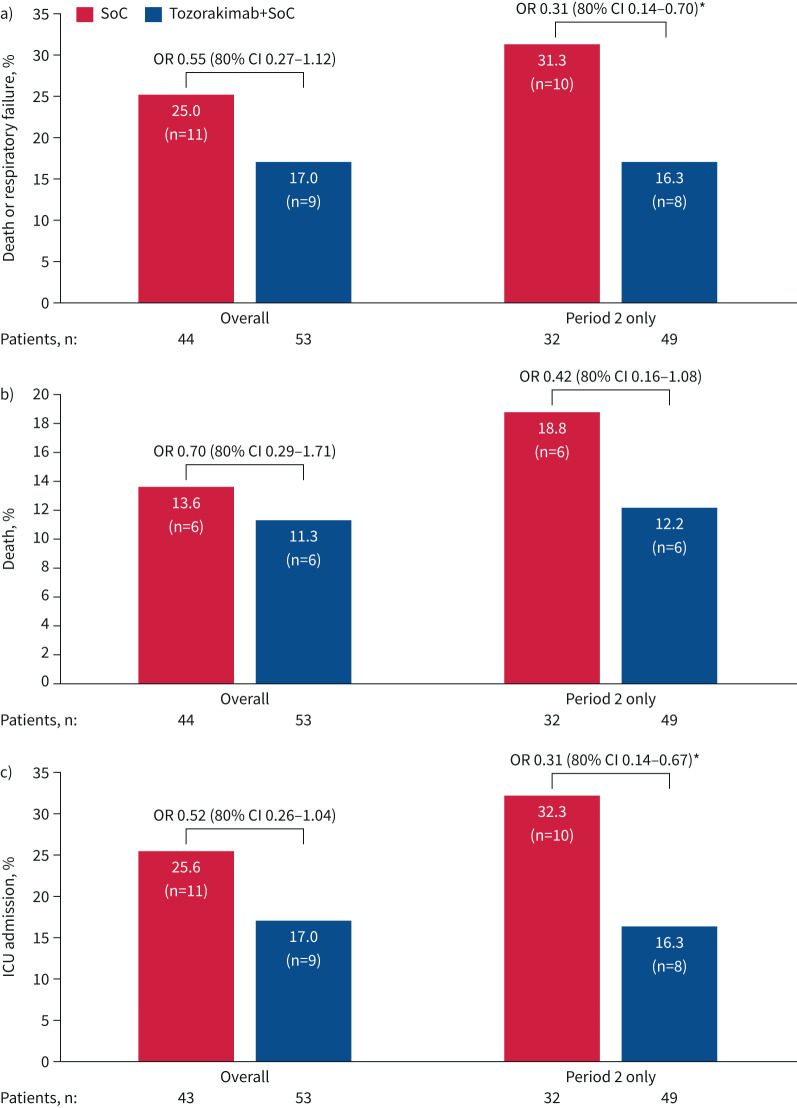
Secondary outcomes overall and in period 2. a) Death or respiratory failure by day 29, b) death and c) intensive care unit (ICU) admission by day 29. One patient in the standard-of-care (SoC) group was excluded from the analysis in c) because they were already in an ICU at the time of randomisation. Percentages are out of the number of patients included in the analysis. Odds ratios (logistic regression model adjusted for age and baseline severity) are given with 80% confidence intervals. *: p<0.1 (one-sided).

20 patients died or had respiratory failure by day 29: nine (17.0%) in the tozorakimab arm and 11 (25.0%) in the SoC arm (figure 3a). The OR for risk of death or respiratory failure at day 29 with tozorakimab compared with SoC was 0.55 (80% CI 0.27–1.12; p=0.26) (figure 3a).

Mortality at all time-points for the tozorakimab and SoC arms is summarised in supplementary table S2; at day 29, mortality of 11.3% *versus* 13.6% was observed, respectively (OR 0.70, 80% CI 0.29–1.71; p=0.62) (figure 3b).

The proportion of patients who did not deteriorate, as measured by OS score, in the tozorakimab and SoC arms for all time-points is presented in supplementary table S3. The proportion of patients discharged from hospital was 83.0% (n=44) in the tozorakimab arm *versus* 79.5% (n=35) in the SoC arm (HR 0.92, 80% CI 0.68–1.25; p=0.79) (supplementary table S4). The mean±sd proportion of days on ventilation for patients in the tozorakimab and SoC arms was 7.7±21.3% *versus* 12.4±26.0%, respectively (supplementary table S5). By day 29, nine patients (17.0%) in the tozorakimab arm and 11 patients (25.6%) in the SoC arm had been admitted to the ICU (OR 0.52, 80% CI 0.26–1.04) (figure 3c).

### Safety

Treatment-emergent adverse events (TEAEs) of any grade occurred in 39 patients (72.2%) in the tozorakimab arm and in 26 patients (59.1%) in the SoC arm (table 3). The most common TEAE categories (reported in >15% of patients in both groups) in the tozorakimab and SoC arms, respectively, were infections and infestations (20.4% and 25.0%), respiratory, thoracic and mediastinal disorders (22.2% and 20.5%), and gastrointestinal disorders (20.4% and 15.9%).

**TABLE 3:** Treatment-emergent adverse events (TEAEs) and serious TEAEs in the safety analysis set

	**Tozorakimab+SoC** **(n=54)**	**SoC** **(n=44)**
**TEAEs**	39 (72.2)	26 (59.1)
System organ class		
Infections and infestations	11 (20.4)	11 (25.0)
Respiratory, thoracic and mediastinal disorders	12 (22.2)	9 (20.5)
Gastrointestinal disorders	11 (20.4)	7 (15.9)
Investigations	11 (20.4)	4 (9.1)
Nervous system disorders	5 (9.3)	8 (18.2)
Cardiac disorders	7 (13.0)	5 (11.4)
General disorders and administration site conditions	2 (3.7)	8 (18.2)
Metabolism and nutrition disorders	4 (7.4)	6 (13.6)
Psychiatric disorders	4 (7.4)	5 (11.4)
Skin and subcutaneous tissue disorders	5 (9.3)	3 (6.8)
Musculoskeletal and connective tissue disorders	4 (7.4)	4 (9.1)
Renal and urinary disorders	5 (9.3)	1 (2.3)
Preferred terms occurring in three or more patients in either arm		
Pulmonary embolism	3 (5.6)	5 (11.4)
Subcutaneous emphysema	0 (0.0)	3 (6.8)
Atrial fibrillation	2 (3.7)	4 (9.1)
Delirium	2 (3.7)	3 (6.8)
Dyspnoea	4 (7.4)	2 (4.5)
Epistaxis	5 (9.3)	0 (0.0)
Oral candidiasis	1 (1.9)	4 (9.1)
Constipation	4 (7.4)	0 (0.0)
Diarrhoea	1 (1.9)	3 (6.8)
Fall	3 (5.6)	1 (2.3)
Pneumonia	1 (1.9)	3 (6.8)
Increased alanine aminotransferase	3 (5.6)	0 (0.0)
**Serious TEAEs occurring in two or more patients in either arm**		
Patients with serious TEAEs	14 (25.9)	10 (22.7)
Pulmonary embolism	2 (3.7)	3 (6.8)
Dyspnoea	3 (5.6)	0 (0.0)
Sepsis	2 (3.7)	1 (2.3)
Acute myocardial infarction	2 (3.7)	0 (0.0)
COVID-19	2 (3.7)	0 (0.0)
Pneumonia	1 (1.9)	1 (2.3)
**TEAEs leading to death**		
Patients with TEAEs leading to death	2 (3.7)	4 (9.1)
Acute respiratory distress syndrome	0 (0.0)	1 (2.3)
Blood culture positive	0 (0.0)	1 (2.3)
Carotid artery occlusion	0 (0.0)	1 (2.3)
Catheter site haemorrhage	1 (1.9)	0 (0.0)
Cerebral artery occlusion	0 (0.0)	1 (2.3)
Cerebral haemorrhage	1 (1.9)	0 (0.0)
Cerebral infarction	0 (0.0)	1 (2.3)
General physical health deterioration	0 (0.0)	1 (2.3)
* Klebsiella* infection	1 (1.9)	0 (0.0)
Multiple organ dysfunction syndrome	0 (0.0)	1 (2.3)
Pulmonary embolism	0 (0.0)	1 (2.3)
Sepsis	1 (1.9)	0 (0.0)
Bacterial superinfection	0 (0.0)	1 (2.3)

Data are presented as n (%). SoC: standard of care; COVID-19: coronavirus disease 2019.

Serious TEAEs of any grade occurred in 14 patients (25.9%) in the tozorakimab arm and in 10 patients (22.7%) in the SoC arm. The most common serious TEAEs in the tozorakimab and SoC arms, respectively, were pulmonary embolism (3.7% and 6.8%), dyspnoea (5.6% and 0.0%) and sepsis (3.7% and 2.3%). TEAEs leading to death occurred in two patients (3.7%) in the tozorakimab arm and four patients (9.1%) in the SoC arm (table 3).

### *Post hoc* analyses

In the subgroup of patients recruited in period 2, there was a statistically significant reduction in the risk of death or respiratory failure at day 29 in those treated with tozorakimab compared with SoC (8/49 (16.3%) *versus* 10/32 (31.3%), respectively; OR 0.31, 80% CI 0.14–0.70) (figure 3a). In this subgroup, mortality of 12.2% *versus* 18.8% in the tozorakimab and SoC arms, respectively, was observed by day 29 (OR 0.42, 80% CI 0.16–1.08) (figure 3b). The risk of ICU admission was also significantly reduced in the tozorakimab arm compared with the SoC arm in this subgroup (OR 0.31, 80% CI 0.14–0.67) (figure 3c).

### Exploratory biomarker analyses

The secondary outcome of risk of death or respiratory failure at day 29 was assessed by baseline levels of the serum biomarker IL-33/sST2 complex. In this *post hoc* exploratory analysis, patients were divided into two groups based on the median baseline level of IL-33/sST2 (<30.15 and ≥30.15 U·mL^−1^).

**FIGURE 4:**
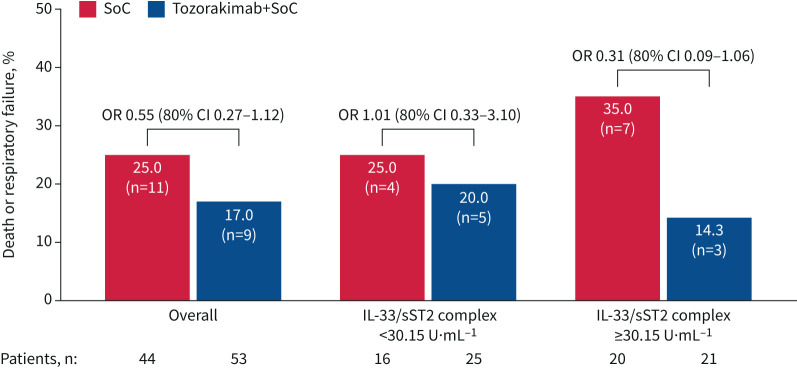
Death or respiratory failure at day 29 by baseline level of serum interleukin (IL)-33/soluble stimulated-2 (sST2) complex. Some patients had missing biomarker values at baseline. The cut-off is the median baseline IL-33/sST2 complex value. Odds ratios were calculated from a logistic regression model adjusting for age and baseline severity. SoC: standard of care.

A total of 41 patients had IL-33/sST2 levels <30.15 U·mL^−1^ (tozorakimab arm, n=25; SoC arm, n=16). Among these patients, five (20.0%) in the tozorakimab arm and four (25.0%) in the SoC arm had experienced death or respiratory failure by day 29. There was no difference in the risk of death or respiratory failure between the treatment arms in this subgroup (OR 1.01, 80% CI 0.33–3.10) (figure 4).

A total of 41 patients had IL-33/sST2 levels ≥30.15 U·mL^−1^ (tozorakimab arm, n=21; SoC arm, n=20). Among these patients, three (14.3%) in the tozorakimab arm and seven (35.0%) in the SoC arm had experienced death or respiratory failure by day 29. The OR for risk of death or respiratory failure at day 29 with tozorakimab compared with SoC in this subgroup was 0.31 (80% CI 0.09–1.06) (figure 4).

The risk of death or respiratory failure at day 29 was also assessed using baseline serum sST2 levels, with the median baseline level (114.6 ng·mL^−1^) used as a cut-off point. The OR for risk of death or respiratory failure at day 29 with tozorakimab compared with SoC was 0.62 (80% CI 0.15–2.58) in the group of patients with baseline serum sST2 levels below the median level and 0.44 (80% CI 0.17–1.10) in those with baseline sST2 levels at or above the median level (supplementary figure S1).

## Discussion

In this study, the primary end-point (time to clinical response) was similar between the tozorakimab and SoC arms. Although the addition of tozorakimab to SoC in patients hospitalised with COVID-19 led to no significant overall improvement in clinical measures, tozorakimab treatment numerically reduced the risk of death, respiratory failure and ICU admission compared with SoC alone. Notably, *post hoc* analysis suggests that tozorakimab treatment may have enhanced effects in a subgroup of patients with higher baseline levels of serum IL-33/sST2.

In this study, the recruitment of patients occurred during two periods, with most patients enrolled in period 2. Of note, during period 2 there were statistically significant reductions with tozorakimab *versus* SoC alone in the proportion of patients who died or had respiratory failure and in the proportion of patients admitted to an ICU. Period 1 occurred before the emergence of the B.1.1.7 virus, which was more virulent than previous COVID-19 variants [22]. Therefore, the severity of COVID-19 in patients enrolled during period 1 may not reflect the disease severity of those currently being admitted to hospital with COVID-19 (*i.e.* those at high risk of severe disease or immunocompromised individuals). For example, during period 1 there were no deaths, two patients presented with respiratory failure and only two ICU admissions occurred. Additionally, dexamethasone was included as the SoC in period 2, whereas period 1 mainly occurred before the use of dexamethasone. Therefore, data from period 2 are likely to be more relevant to current clinical practice than period 1. Overall, these results highlight the possibility that tozorakimab could be an effective therapy for patients hospitalised with COVID-19 who are at risk of acute respiratory failure or death, even in conjunction with dexamethasone therapy.

Targeting the IL-33/ST2 axis has shown potential for controlling excessive lung inflammation [27]; several phase 2 trials are investigating anti-ST2 and anti-IL-33 antibodies as therapies for other inflammatory diseases, such as COPD (ClinicalTrials.gov: NCT03546907 and NCT03615040) and asthma (ClinicalTrials.gov: NCT03207243) [28]. An increased understanding of key mechanisms that drive poor outcomes in patients with COVID-19 has facilitated the development of potential therapeutic strategies targeting the aberrant host hyper-inflammatory response [3, 29, 30]. IL-33 represents an attractive therapeutic target for COVID-19 because increased IL-33 levels might facilitate excess lung inflammation in patients hospitalised with COVID-19 and serum IL-33 levels correlate with poor clinical outcomes [31, 32].

Identification of precision medicine biomarkers may help to stratify patients to specific treatments that improve patient outcomes in COVID-19. In this study, exploratory biomarker analysis indicated that tozorakimab may have a greater benefit in patients with elevated baseline levels of the IL-33/sST2 complex. We hypothesise that higher levels of circulating IL-33/sST2 complex reflect increased release of IL-33 in the airways and that tozorakimab, by neutralising IL-33, will have a greater benefit in this population. Consistent with this hypothesis, patients with high levels of sST2 did not preferentially benefit from tozorakimab. However, further studies of the interplay of IL-33 and sST2 and the mechanism of tozorakimab in patients with COVID-19 are required.

Data from this study confirmed that tozorakimab was well tolerated in patients hospitalised with COVID-19. A higher proportion of patients in the tozorakimab arm experienced TEAEs than in the SoC arm; however, patients receiving active treatment in an open-label study compared with a blinded study may report a greater number of adverse events than those receiving SoC [33]. Of note, the proportion of patients with serious TEAEs was similar in both groups. Therefore, no safety findings from this study preclude further development of tozorakimab.

The health and economic impact of COVID-19 has been substantial [34], and a treatment that reduces mortality could save a significant number of lives and reduce the burden on healthcare systems. Dexamethasone has been shown to reduce mortality in patients with COVID-19 needing oxygen and ventilation by 18% and 36%, respectively [35]. In our study, treatment effects were observed in patients with severe disease and those with high baseline levels of serum IL-33/sST2 complex treated with both tozorakimab and SoC *versus* SoC alone. Consequently, tozorakimab could reduce mortality more than SoC alone, further alleviating the burden on healthcare systems.

Limitations of this study include the small sample size, particularly in subgroup analyses. The target sample size (≥54 patients per arm) was not reached owing to recruitment challenges related to the rapidly changing environment of the COVID-19 pandemic. This led to a pause in recruitment after the end of the first UK COVID-19 wave. Conducting a study in an intense hospital environment meant that complete baseline data were not always available. Patients in the tozorakimab arm had a higher mean baseline NEWS2 score and a greater number of comorbidities than those in the SoC arm, suggesting that they had a worse prognosis. SoC changed during the study; the use of dexamethasone and tocilizumab was more widespread in period 2. Thus, the current SoC received by patients hospitalised with COVID-19 may differ from the SoC used in this study, which may limit the generalisability of the results. Furthermore, the emergence of COVID-19 variants during the study, which was conducted before widespread vaccination, should be considered when interpreting these results. In line with this, most patients who were included in the study were unvaccinated. Owing to the subsequent vaccination programme in the UK, patients who are hospitalised with COVID-19 in current practice are likely to have increased immunity compared with the patients who were assessed during this study. Finally, the sensitivity of the primary end-point was limited because some patients recovered quickly from COVID-19, which may diminish the actual benefit observed in those with severe outcomes.

### Conclusions

Results from this study demonstrated that tozorakimab was well tolerated. The primary end-point was similar between the tozorakimab and SoC arms, and tozorakimab showed no significant effect in reducing the risk of respiratory failure and death, or in reducing ICU admissions, compared with SoC overall. However, findings that the treatment effect of tozorakimab may be enhanced in a subgroup of patients recruited during a period associated with more severe disease and in those with high baseline serum IL-33/sST2 complex levels warrant further investigation. A global phase 3 study is underway to assess the efficacy of tozorakimab in patients hospitalised with viral lower respiratory tract disease (TILIA; ClinicalTrials.gov: NCT05624450).

## Supplementary material

10.1183/23120541.00249-2023.Supp1 **Please note:** supplementary material is not edited by the Editorial Office, and is uploaded as it has been supplied by the author.
Supplementary material 00249-2023.SUPPLEMENT
