# Supplementary material for: A randomised phase 2a study to investigate the effects of blocking interleukin-33 with tozorakimab in patients hospitalised with COVID-19: ACCORD-2
Source: ERJ Open Res. 2023 Oct 2;9(5):00249-2023. doi: 10.1183/23120541.00249-2023 (PMC10588785; doi:10.1183/23120541.00249-2023)
Supplement: Supplementary file 1 [file 00249-2023.SUPPLEMENT.pdf]

Supplementary appendix

Supplementary methods

Study design and participants

Patient enrolment for each agent arm continued until the planned enrolment target was achieved.

Inclusion and exclusion criteria

Inclusion criteria

1. Adults (aged ≥18 years) with severe acute respiratory syndrome coronavirus (SARS-CoV-2) infection confirmed by laboratory tests and/or point of care tests.
2. A score of 3–5 on the 9-point World Health Organization ordinal scale.
3. The patient and their partner agreed to use medical-accepted double-barrier methods of contraception (*e.g.* barrier methods, including male condom, female condom or diaphragm with spermicidal gel) during the study and for at least 6 weeks after termination of study therapy. Having a vasectomised partner was considered an appropriate birth control method provided that the partner was the sole male sexual partner and the absence of sperm had been confirmed. If not, an additional method of contraception was used, or the patient was a woman who was not of childbearing potential.
4. Women who were lactating who agreed not to breastfeed their child during the study and for at least 6 weeks after termination of study therapy (they could continue to express milk away from the child during this period, but this milk must be discarded).
5. Ability to provide informed consent signed by the study patient or legally authorised representative.

Exclusion criteria

Patients were excluded from the study is any of the following criteria applied (or any of the criteria from the appropriate sub-protocol).

1. Patients who previously had a score of 6 or 7 on the 9-point ordinal scale.
2. Any patients whose interests were not best served by study participation as determined by a senior attending clinician.
3. Alanine aminotransferase/aspartate aminotransferase >5 x the upper limit of normal.
4. Known active infection with human immunodeficiency virus or hepatitis B or C.

5. Stage 4 severe chronic kidney disease or requiring dialysis (*i.e.* estimated glomerular filtration rate <30 mL/min/1.73 m^2).
6. History of the following cardiac conditions:
   a. myocardial infarction within 3 months prior to the first dose
   b. unstable angina
   c. history of clinically significant dysrhythmias (long QT features on electrocardiogram, sustained bradycardia [≤55 bpm]), left bundle branch block, cardiac pacemaker or ventricular arrhythmia) or history of familial long QT.
7. Screening 12-lead electrocardiogram with a measurable QTc interval according to Fridericia correction >500 ms.
8. Anticipated transfer to another hospital that was not a study centre within 72 hours.
9. Allergy to any study medication.
10. Experimental off-label usage of medicinal products as treatments for coronavirus disease 2019 (COVID-19).
11. Patients participating in another clinical study of an investigational medicinal product.
12. Active tuberculosis defined as requiring current treatment for tuberculosis.

Randomisation

Patients were randomly assigned to receive treatment with equal probability of randomisation to each of the study arms running at the study site at the time of enrolment. The study was open label, and both investigators and patients were aware of treatment allocation. The randomisation ratio was automatically adjusted, accounting for the number of study arms available at each study site, to ensure that the number of patients randomised to each candidate agent plus standard of care (SoC) was approximately equal to the number randomised to SoC alone. Patients were excluded from randomisation to a candidate agent if they did not meet the eligibility criteria specified in the sub-protocol. The allocation sequence was generated by Cenduit Interactive Response Technology with the electronic case report form assigned a unique randomisation number, linked to a treatment arm, to the patients. Randomisation was stratified by study centre and baseline disease severity grade.

Procedures

Based on phase 1 clinical pharmacokinetics data, a single 300 mg intravenous dose of tozorakimab was predicted to suppress IL-33 levels more than 99% at peak drug concentration in sputum. The optimal serum concentration for tozorakimab efficacy in patients with COVID-19 was not known at the start of enrolment. Rationale for administration of a second dose 14 days after the first dose was based on the approximate terminal half-life

of tozorakimab and the results of a 4-week toxicology study, which predicted a highly favourable safety margin (>47-fold) when a second dose of tozorakimab 300 mg was administered at this interval. SoC treatment in both study arms continued until hospital discharge. Administration of the second dose of tozorakimab when it would otherwise be required did not occur for the following reasons: patient request, protocol violation, any clinically significant adverse event (AE), any serious AE (SAE), severe laboratory test abnormality, pregnancy or deemed not to be in the best interest of the patient by the investigator.

SoC

Patients were enrolled by study site investigators and randomised to receive one of the candidate treatments being evaluated at that time or the SoC arm. SoC treatment during the study could include oral once-daily remdesivir for 5 days, once-daily dexamethasone until hospital discharge, or tocilizumab on top of dexamethasone, and respiratory support with supplemental oxygen or non-invasive ventilation. At each site, patients could receive either SoC or a candidate agent; therefore, the SoC arm was shared between the candidate experimental arms in the study.

Data for all treatment-emergent adverse events (TEAEs) and serious TEAEs were collected regardless of causality; events were managed according to physician judgement and applicable national guidelines. All serious TEAEs were followed until resolution, stabilisation or event explanation (if the patient was not lost to follow-up). Adverse events were graded according to National Cancer Institute Common Terminology Criteria for Adverse Events, version 5.0, and were coded using the Medical Dictionary for Regulatory Activities, version 24.0. All SAEs were reported by the investigator to the sponsor within 24 hours of identification. COVID-19-related events that met the definition of SAEs did not require expedited reporting. Cardiovascular, renal and liver organ failure were reported as AEs of special interest in both study arms and serious hypersensitivity, hepatic function abnormality, cardiac events, serious infections, serious gastrointestinal events and malignancies were reported as Aes of special interest in the tozorakimab arm. In both study arms, clinical status, Aes, vital signs (body temperature, pulse, blood pressure, respiratory rate, oxygen saturation), concomitant medications and survival were monitored at baseline and daily until hospital discharge, and at day 15 and 29 if the patient was discharged before day 15. Blood gases (fraction of inspired oxygen and partial pressure of oxygen) followed the same schedule excluding day 29 if the patient had already been discharged.

The last day of these assessments while hospitalised was day 29. Physical examination was performed at screening and daily until hospital discharge (focusing on lung auscultation during treatment). Clinical status was assessed by the ordinal scale score, National Early Warning Score 2 oxygen requirement and non-invasive or invasive ventilator requirement. Laboratory safety assessments (haematology, blood chemistry, liver function, coagulation) were performed at screening, at baseline and on days 3, 5, 8 and 11 while patients were hospitalised. Laboratory research assessments were performed at baseline and on day 5 and day 10 for inflammatory cytokine biomarker analysis and host transcriptome analyses, and at baseline and on day 15 for host serological SARS-CoV-2 response analysis. In the tozorakimab arm, further laboratory assessments were performed for pharmacokinetic and immunogenicity analyses.

The protocol only mandated laboratory safety assessments. SARS-CoV-2 infection was monitored by reverse transcription polymerase chain reaction from nasopharyngeal swab at baseline and on days 3, 5, 8, 11, 15 and 29. Study follow-up for Aes and survival occurred as outpatient visits at days 60 and 90. The aim was to perform all assessments unless the patient withdrew consent or was lost to follow-up.

Statistical analysis

Primary analysis was conducted in patients who were randomised, had at least one post-baseline ordinance scale score and received at least one dose of study medication (efficacy population). The primary method of statistical comparison was a stratified log-rank test.

Ties were handled using the exact method. Confidence intervals were calculated according to the Brookmeyer and Crowley method. There was no imputation of missing data.

Summary statistics and shift tables were generated for additional secondary endpoints. No adjustments for multiple testing were made.

All safety endpoints were evaluated in patients who underwent randomisation and received at least one dose of study medication, regardless of recording a post-baseline ordinal score (safety analysis set).

Independent data and safety monitoring committee

An independent data and safety monitoring committee was established to assess safety on an ongoing basis throughout the study. This committee held a formal review halfway through each recruitment period.

SUPPLEMENTARY TABLE 1 Baseline demographics and patient characteristics of patients enrolled in period 2 in the safety analysis set[a]

Demographic/characteristic	Tozorakimab + SoC (n=50)	SoC (n=32)
**Age, years, mean (SD)**	56.1 (12.4)	56.9 (13.7)
Age ≥70 years	8 (16.0)	6 (18.8)
**Sex, male**	34 (68.0)	21 (65.6)
**Body mass index, kg/m^2, mean (SD)**	31.6 (7.4)	32.7 (7.8)
**Smoking status[b]**		
Former	25 (50.0)	14 (45.2)
Current	2 (4.0)	0 (0.0)
**Time since onset of symptoms**		
<12 days	34 (68.0)	27 (84.4)
≥12 days	16 (32.0)	5 (15.6)
**Derived baseline WHO OS score**		
Grade 3	1 (2.0)	0 (0.0)
Grade 4	38 (76.0)	26 (81.3)
Grade 5	11 (22.0)	6 (18.8)
**NEWS2 score, mean (SD)**	4.7 (2.3)	4.1 (1.6)
**Clinical frailty score at baseline**		
Very fit	8 (16.0)	4 (12.5)
Well	21 (42.0)	13 (40.6)
Managing well	8 (16.0)	4 (12.5)
Vulnerable	4 (8.0)	1 (3.1)
Mildly frail	2 (4.0)	3 (9.4)
Moderately frail	7 (14.0)	7 (21.9)
**Comorbidities at baseline**		
≥1	32 (64.0)	18 (56.3)
≥2	20 (40.0)	9 (28.1)
**Comorbidity categories**		
Heart disease	5 (10.0)	3 (9.4)
Diabetes	20 (40.0)	10 (31.3)
Chronic lung disease	7 (14.0)	2 (6.3)
Chronic liver disease	1 (2.0)	0 (0.0)
Asthma[b]	7 (14.0)	5 (16.1)

Tuberculosis	0 (0.0)	1 (3.1)
Cancer	5 (10.0)	1 (3.1)
Hypertension	15 (30.0)	9 (28.1)
**Remdesivir at baseline**	32 (64.0)	22 (68.8)
**Dexamethasone at baseline**	50 (100.0)	31 (96.9)
**Supplemental oxygen at baseline**	49 (98.0)	32 (100.0)
**Received COVID-19 vaccine during the study**	13 (26.0)	4 (12.5)

Data are presented as n (%) unless stated otherwise. [a]Percent values are based on the safety analysis set. One patient in the tozorakimab arm did not have a post-dose OS assessment and was excluded from the full analysis set but included in the safety analysis set. [b]Percent values are based on 50 patients receiving tozorakimab and 31 patients receiving SoC. COVID-19: coronavirus disease 2019; HIV: human immunodeficiency virus; NEWS: National Early Warning Score; OS: ordinal scale; SD: standard deviation; SoC: standard of care; WHO: World Health Organization.

SUPPLEMENTARY TABLE 2 Mortality at days 15, 29 and 60

	**Tozorakimab + SoC (N=53)**	**SoC (N=44)**	**P value**
**Day 15**			
Mortality, n (%)	3 (5.7)	4 (9.1)	
Odds ratio (80% CI)	0.45 (0.14–1.39)	NA	0.42
**Day 29**			
Mortality, n (%)	6 (11.3)	6 (13.6)	
Odds ratio (80% CI)	0.70 (0.29–1.71)	NA	0.62
**Day 60**			
Mortality, n (%)	8 (15.1)	9 (20.5)	
Odds ratio (80% CI)	0.60 (0.27–1.33)	NA	0.38

CI: confidence interval; NA: not available; SoC: standard of care.

SUPPLEMENTARY TABLE 3 Non-deterioration in OS score on days 15 and 29

	Tozorakimab + SoC (N=53)	SoC (N=44)	P value
**Day 15**			
No deterioration by at least 1 point on the OS, n (%)	45 (84.9)	34 (77.3)	
Odds ratio (80% CI)	1.83 (0.89–3.76)	NA	0.27
**Day 15**			
No deterioration by at least 2 points on the OS, n (%)	46 (86.8)	35 (79.5)	
Odds ratio (80% CI)	1.77 (0.85–3.67)	NA	0.30
**Day 15**			
No deterioration by at least 3 points on the OS, n (%)	48 (90.6)	40 (90.9)	
Odds ratio (80% CI)	1.04 (0.40–2.70)	NA	0.97
**Day 29**			
No deterioration by at least 1 point on the OS, n (%)	44 (83.0)	33 (75.0)	
Odds ratio (80% CI)	1.81 (0.89–3.68)	NA	0.26
**Day 29**			
No deterioration by at least 2 points on the OS, n (%)	45 (84.9)	33 (75.0)	
Odds ratio (80% CI)	2.07 (1.02–4.21)	NA	0.18
**Day 29**			
No deterioration by at least 3 points on the OS, n (%)	47 (88.7)	36 (81.8)	
Odds ratio (80% CI)	2.02 (0.88–4.64)	NA	0.27

CI: confidence interval; NA: not available; OS: World Health Organization ordinal scale; SoC: standard of care.

SUPPLEMENTARY TABLE 4 Time to sustained live discharge from hospital

	**Tozorakimab + SoC (N=53)**	**SoC (N=44)**	**P value**
**Patients with sustained live discharge from hospital, n (%)**	44 (83.0)	35 (79.5)	
**Patients with censored data, n (%)**	9 (17.0)	9 (20.5)	
**Median, days**	8.0	9.5	
**HR (80% CI)**	0.92 (0.68–1.25)	NA	0.79

CI: confidence interval; HR: hazard ratio; NA: not available; SoC: standard of care.

SUPPLEMENTARY TABLE 5 Duration of ventilation use

	**Tozorakimab + SoC (N=53)**	**SoC (N=44)**
**Duration of ventilation use, days**	1.8 (5.3)	3.1 (7.2)
**Percentage of days on ventilation**	7.7 (21.3)	12.4 (26.0)
**Number of ventilation-free days**	25.6 (8.0)	24.0 (9.3)
**Percentage of ventilation-free days**	92.3 (21.3)	87.6 (26.0)

Data are mean (standard deviation). SoC: standard of care.

SUPPLEMENTARY FIGURE 1 Death or respiratory failure at day 29 by baseline level of sST2.

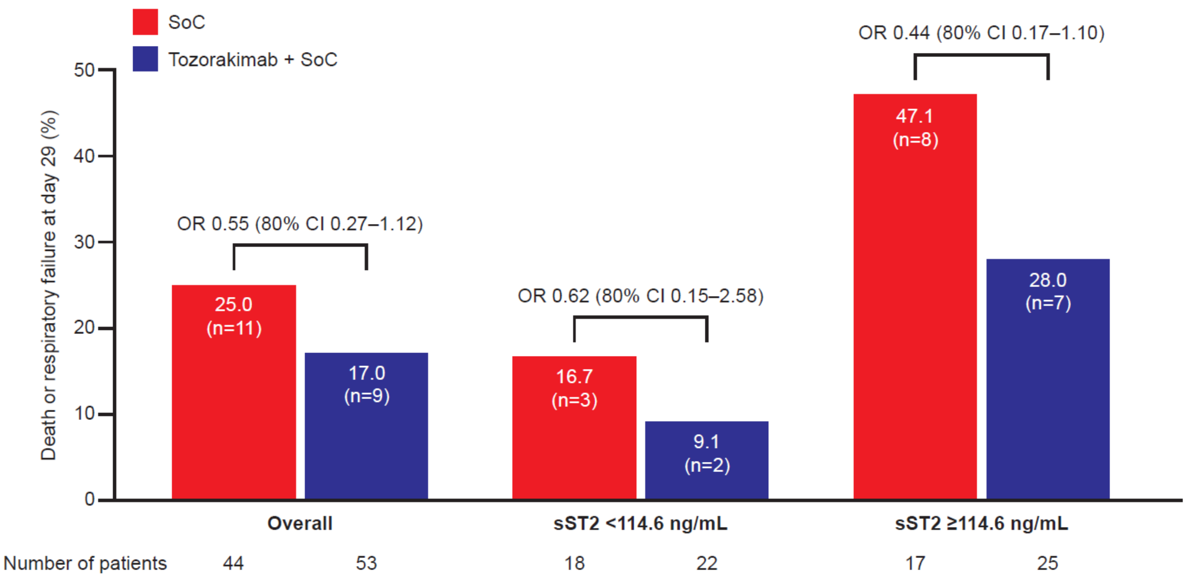

Some patients had missing biomarker values at baseline. The cut-off is the median baseline sST2 value. RR was calculated directly from observed proportions, without adjustment for other factors. OR was calculated from a logistic regression model adjusting for age and baseline severity. CI: confidence interval; OR: odds ratio; SoC: standard of care; sST2: soluble ST2.
